# Perceptions of Caring Behaviors among Patients and Nurses

**DOI:** 10.3390/ijerph20010396

**Published:** 2022-12-27

**Authors:** Victoria Alikari, Georgia Gerogianni, Evangelos C. Fradelos, Martha Kelesi, Evridiki Kaba, Sofia Zyga

**Affiliations:** 1 Department of Nursing, University of Peloponnese, 22100 Tripoli, Greece; 2 Department of Nursing, University of West Attica, 12243 Athens, Greece; 3 Department of Nursing, University of Thessaly, 41500 Larissa, Greece

**Keywords:** perceptions, caring behaviors, patients, nurses

## Abstract

The concept of caring is fundamental to nursing practice. The aim of this study was to investigate patients’ and nurses’ caring behaviors and the possible differences between the two groups. In this descriptive and comparative study, 310 patients and 329 nurses from six general hospitals from Greece completed the Caring Behaviors Inventory-16. The mean score of Caring Behaviors Inventory-16 for patients was 78.94 (±17.85) and for nurses 80.27 (±9.36). The items “Demonstrating professional knowledge and skills” (Mean: 5.45 ± 3.62) and “Treating my information confidentially” (Mean: 5.34 ± 1.06) were the most important caring behaviors while the items “Including me in planning care” (Mean: 4.36 ± 1.56), and “Treating me as an individual” (Mean: 4.55 ± 1.46) were the least important caring behaviors for patients. For nurses, the most important caring behavior was “Treating patients” information confidentially” (Mean: 5.43 ± 0.94) and the least important was “Returning to the patient voluntarily” (Mean: 4.57 ± 3.68). Significant differences were observed in items: “Attentively listening to me/the patient” (*t* = −2.05, *p* = 0.04), “Treating me/the patient as an individual” (*t* = −7.82, *p* = 0.00), “Being empathetic or identifying with me/the patient” (*t* = −2.80, *p* = 0.00), and “Responding quickly when I/the patient call (*t* = −2.01, *p* = 0.04). Respect, privacy, and dignity were the most important caring behaviors for nurses while for patients they were knowledge, skills, and safety.

## 1. Introduction

Caring is a fundamental element of nursing practice adopted by Florence Nightingale in the early 1800s. It is universally accepted that nurses care for individuals in their experience of illness [1]. Caring is determined by the values and the ethical professional code that nurses incorporate into their practice [2]. It is described as a human trait, moral obligation, interpersonal relationship, and therapeutic intervention. Theories of caring typically are classified into two general categories of caring behaviors: those that reflect the skills or technical abilities and those that reflect the attitudes and behaviors or emotional aspects of caring. The concept of caring was defined as the manifestation of compassion, concern for others, kindness, human interaction, affection, interpersonal relationship, and therapeutic alliance [3].

In addition to the aforementioned caring activities, nurses also express behaviors that are not expressed through the clinical nursing activities provided. Nurses play a role in protecting the patient’s privacy and respecting the patient’s dignity, values, and beliefs. This is a characteristic of each nurse separately that influences caring behaviors [4]. Nurses, through effective communication with patients, contribute to the development of a therapeutic relationship with the patient thus facilitating the expression of patients’ emotions. According to Kruijver [5], attendance, touch, and listening are elements of caring that help patients externalize their feelings [5]. Presence does not only refer to the physical presence of the nurse but also the organization of the care plan [6]. Touch is associated not only with the performance of clinical interventions on the patient’s body but also with emotional communication (therapeutic touch) through which the patient’s self-confidence is stimulated [7]. The concept of listening is expressed not only by the biological ability of hearing, but also by the eye-contact and facial expressions [8]. Thus, nurses, through these caring behaviors, promote self-esteem and the process of adaptation to illness [9].

When practicing person-centered care, treatment recommendations, and clinical decisions respect and consider patients’ preferences, beliefs, and values [10]. The need to face this situation and perform person-centered behaviors finds support in Jean Watson’s theory [11], according to which caring is an interrelated process of shared feelings between the nurse and the patient. According to Watson, caring includes values, the commitment to care, knowledge, caring actions, and, also, a commitment to alleviate the vulnerability of the patient, providing care and concern for every human life. This provision of caring is characterized by Watson as a moral ideal [11]. Watson defined caring as “the process by which the nurse becomes responsive to another person as a unique individual, perceives the others’ feelings, and sets that person apart” [12]. There are also many nurses who equate care with understanding. Understanding in this particular case means understanding the demands, wants, and needs of a person who needs care. In this case, the demand for emotional understanding is highlighted, especially for those people who cannot express their feelings due to their medical condition [13].

Studies that aim to compare patient’s and nurse’s perceptions of care have revealed both similarities and differences and it seems that the type of the department is an important differentiating factor in perceptions of caring behaviors. Particularly, in a study [14] conducted in an emergency department, the most important caring behavior for patients is the clinical competence followed by preservation of their individuality [14] while in another study [15] conducted in various clinical departments (of internal medicine, of surgery, of urology), patients and nurses similarly emphasize the aspect of needs’ satisfaction though patients refer to the highest levels of this dimension [15]. On the other hand, nurses perceive their psychological skills and expressive or emotional caring behavior as more important than patients [16]. Patients from different cultures describe the “good” nurse in a similar way [17]. This means that regardless of the cultural background, patients ask nurses to have the same caring behaviors and in particular to have scientific knowledge and ability, but also to strengthen their interpersonal relationships [18].

In summary, the evidence regarding the perception of caring by patients and nurses is ambiguous and needs to be clarified to enhance the care required. The different variations of the concept of caring and the perceptions of it necessitated the development of this research. Therefore, the aim of this study was to explore the differences in caring behaviors between nurses and patients.

## 2. Materials and Methods

### 2.1. Study Design

This is a cross-sectional study with a comparative analysis.

### 2.2. Settings

The study was conducted in three hospitals in Athens and Thessaloniki (the most densely populated regions of Greece) and three hospitals in the Peloponnese region (Kalamata, Kyparissia, and Korinthos). The above hospitals are public general hospitals.

### 2.3. Study Sample

The sample consisted by nurses working and patients hospitalized in the above hospitals. The inclusion criteria for the sample of nurses were: (i) to be clinical nurses/assistant nurses of the hospitals with at least 1 year of work experience; (ii) to read and speak the Greek language fluently; (iii) not to be nursing students and nurses with eye or mental disorders. Of the total of 379 nurses, 329 completed the questionnaires (response rate of 86.8%).

Regarding the recruitment of patients, the inclusion criteria were: (i) to be hospitalized patients in one of these 6 hospitals for at least 3 days, (ii) to be adults, and (iii) to read and speak the Greek language fluently. Patients with mental or eye disorders were excluded. Of a total of 412 patients, 315 were eligible. The rest 97 patients did nοt meet the criteria and excluded from the study. Finally, 310 accepted to complete the questionnaires (response rate of 75.2% in the sample of the eligible patients). The questionnaires were distributed by the researchers to both patients and nurses in a closed envelope. The study was carried out during the period from January to March 2021.

### 2.4. Data Collection

Data were collected using the Greek version of the Caring Behaviors Inventory-16 (CBI-16) [19]. CBI-16 is a self-administered anonymous questionnaire revised in 2017 [20] to explore caring behaviors as perceived by patients. The Caring Behaviors Inventory-16 (CBI-16) is derived from Watson’s caring theory and emphasizes nurse-patient interaction in therapeutic relationships [21]. It consists of 16 states rated on a 6-point Likert scale (1 = never to 6 = always). The CBI-16 is the revised form of the CBI-42 and CBI-24. Even though the CBI-24 studies four aspects (“Human Presence”, “Professional Knowledge”, “Respectful Deference”, and “Connectness”) of caring, the Greek version of CBI-16 is unidimensional. The total score can range from 16–96. Higher scores indicate that participants perceive the behaviors as more important. The CBI-16 can also be used among nurses and thus comparisons are possible. The 16-item version has been used in several studies in Saudi Arabia [22], Ethiopia [23], and in Iran [24]. The internal consistency of the Greek version is very good (Cronbach’s Alpha Index 0.967) [19]. Demographic, clinical, and social data for both of the groups were also recorded.

### 2.5. Ethics

Licenses from the Ethical Committees of the Hospitals (General Hospital of Thessaloniki “G. Papanikolaou” prot. number 345/23.9.2019, General Hospital of Korinthos approval number 45/25.10.2019, General Hospital of Kyparissia approval number 4581/9.10.2019, General Hospital of Athens “G. Gennimatas” approval number 41432/27.12.2018, General Hospital of Piraeus “St. Panteleimon” approval number 54493/25.11.2019, General Hospital of Kalamata approval number 16/31.10.2019) were secured. The study was conducted according to the ethical principles of the Declaration of Helsinki for medical research. Participants were informed that their participation is voluntary and anonymous and that their data will be used only for the research. A written consent form was obtained from the participants.

### 2.6. Data Analysis

The descriptive indicators of the variables were analyzed. Basic measures of location and dispersion (mean and standard deviation) as well as absolute frequencies (N) and relative frequencies (%) were used to describe demographic characteristics and the scores of dimensions of the scale CBI-16. To avoid transcription errors, the frequency, the range of values, the mean and the standard deviation of each variable were checked. The Kolmogorov–Smirnov test was performed to check the normality of the data. *t*-test was used for the association between the independent samples of nurses and patients. *P*-values lower than 0.05 were considered as statistically significant results. To perform the data analysis, the software IBM SPSS Statistics version 21 (SPSS Inc., 20, Chicago, IL, USA) was used.

## 3. Results

From the group of patients, 310 individuals participated. Of these, 157 (47.7%) were men. The mean age of patients was 60.8 (SD ± 16.8) years old and 221 (67.2%) were married. The majority (70.5%) were being treated in internal medicine departments, while 52.9% were admitted to the hospital for chronic disease (Table 1).

Regarding nurses (N = 329), the mean age was 49 (±8.10) years old, ranging between 23 and 60 years, 251 were women (21.6%), and the total working experience as clinical nurse was 17.6 (±8.5) years. The majority (57.8%) were married and university graduates (61.1%) (Table 2).

The mean score of CBI-16 for patients was 78.94 (±17.85) and for nurses 80.27 (±9.36). Table 3 summarizes the patients’ and nurses’ caring behaviors mean scores for each item and the ranking of the CBI-16 items in order of importance for both of the groups. According to the results, patients perceived “Demonstrating professional knowledge and skill” (Mean: 5.45 ± 3.62) and “Treating my information confidentially” (Mean: 5.34 ± 1.06) as the most important caring behaviors and “Including me in planning care” (Mean: 4.36 ± 1.56), “Treating me as an individual” (Mean: 4.55 ± 1.46) as the least important caring behaviors. The most important caring behavior for nurses was “Treating patients’ information confidentially” (Mean: 5.43 ± 0.94) and the least important was “Returning to the patient voluntarily” (Mean: 4.57 ± 3.68). Comparing the responses of nurses and patients, some similarities were observed as both groups have ranked the following items in the same order: Rank 3 “Giving my/patient’s treatments and medications on time”, rank 9 “Responding quickly when I/the patient call”, Rank 10 “Supporting me/the patient”. A notable difference regarding the ranking of the items was observed in item 3 “Treating me/the patient as an individual” as the patients rank it as 15th while the nurses rank it as the 2nd most important caring behavior. Moreover, in three out of 16 items, there was agreement between patients and nurses as they rank them in the same hierarchical order (items 5, 14, and 15).

Table 4 gives the differences in caring behaviors between the two groups (*t*-test). Significant differences were observed in four items (about a quarter of the statements) “Attentively listening to me/the patient” (*t* = −2.05, *p* = 0.04), “Treating me/the patient as an individual” (*t* = −7.82, *p* = 0.00), “Being empathetic or identifying with me/the patient” (*t* = −2.80, *p* = 0.00), and “Responding quickly when I/the patient call (*t* = −2.01, *p* = 0.04). Moreover, in these particular statements, nurses rated higher than patients. Patients valued seven items (2, 7, 8, 11, 13, 15, 16) more than nurses without statistically significant differences.

## 4. Discussion

This study explored the patients’ and nurses’ caring behaviors. The study is significant as the lack of understanding of the differences in perceptions between patients and nurses may negatively affect the quality of care and health outcomes.

Eleven items out of the 16 of the CBI-16 had a mean score over five which indicates that for nurses the most caring behaviors are important. In particular, nurses valued more the item “treating patient’s information confidentially” followed by “treating the patient as an individual”, and “giving patient’s treatments and medications on time”. In studies [20,25] that have used previous versions of the CBI, these items were included in the sections of knowledge and skills, respect, and safety. Therefore, it seems that nurses give the highest priority to the humanitarian dimensions of caring, and patient safety issues. A possible explanation for this is that nurses have an increased sense of responsibility towards the patient, but also of safeguarding personal data and dignity. The above concepts represent the professional values of nursing as demonstrated in ethical codes [26]. The nurses’ perceptions of the importance of skills and individuality in caring are supported by researchers [27] who point out that skills, efficiency, and safety are essential prerequisites for the practice of nursing.

Nurses also rank active listening (“attentively listening to the patient”) and empathy (“being empathetic or identifying with the patient”) among the five most important caring behaviors. Research studies [28] conducted in this scientific area have stressed the positive influence of these behaviors on effective nurse-patient communication and the satisfaction of patients’ needs. Active listening creates a climate of mutual trust and a therapeutic relationship based on respect for the individuality of the patient [29].

According to the nurses’ views, among the least important items were “spending time with the patient”, and “giving instructions or teaching the patient”. It seems that the lack of staff and the resulting workload of nursing staff puts the interpersonal relationships between nurses and patients (provision of instructions and devoting time to patients) in second place, resulting in a low score on these questions [30]. In contrast to this finding, in a study [31] in Japan, nurses rated the item “helping the patient not feel dumb by giving the patient adequate information” as the most important. It could be perceived that these differences can be attributed to cultural differences, and different health and nursing care systems. However, despite the difficulties faced by nurses in Greece due to the lack of staff, the importance of patient education cannot be overlooked, as education promotes self-management, adherence to treatment, and patient participation in clinical decision-making [32].

According to the patients’ answers, seven of the 16 items had a mean score above five. Of these items, three describe professional knowledge and skills (“being confident with the patient”, “demonstrating professional knowledge and skill”, “treating patient’s information confidentially”) while two are related to the feeling of safety (“giving patient’s treatments and medications on time”, “relieving patient’s symptoms”). It is interesting to note that patients valued first the item “demonstrating professional knowledge and skills” followed by “treating my information confidentially”, and “giving my treatments and medications on time”. This indicates that patients consider professional knowledge, skills, and personal data management that promote rapid, safe, and supportive recovery to be among the most important indicators of care. This supports the results of other studies [33,34] which rank the dimensions of skills, knowledge, and safety first in the patients’ hierarchy.

The patient’s inclusion in the planning care (“including me in planning care”) and the individualized care (“treating me as an individual”) are prioritized in the last positions of the patients’ classification. Other studies [35,36] have also found that patients’ scores are low on behaviors, such as patient participation in the care plan, and respect for individuality, leading to the fact that, unlike knowledge, some values derived from care are not transferred to recipients.

This survey showed differences in specific caring behaviors between patients’ and nurses’ perceptions. This is an important finding in the understanding of the perceptions of the two groups. The ranking of the item “treating me/the patient as an individual” between the two groups is remarkable since for nurses this is one of the most important behaviors while patients rank it in the last positions. Therefore, the results demonstrated that nurses give more importance to issues, such as respect (“being empathetic or identifying with the patient”, “treating the patient as an individual”), and listening (“attentively listening to the patient”), which they systematically rank as a priority. In this study, it is worth emphasizing that in the items where there are statistically significant differences, nurses show higher scores compared to the patients. Patients choose more technical behaviors and knowledge (“demonstrating professional knowledge and skill”), safety and monitoring issues (“Giving my treatments and medications on time”, “Returning to me voluntarily”). A possible explanation for nurses rating emotional aspects of caregiving highly, is that nurses who care consciously, experience satisfaction from caregiving and draw strength for the most challenging situations in nursing practice [37]. This corresponds to Watson’s theory of care which emphasizes interpersonal relationships between patients and nurses [38].

Studies [35,39,40] generally show significant differences in perceptions between nurses and patients about nursing caring behaviors. The differences in the prioritization of caring behaviors between patients and nurses may be related to the different ways of understanding the various aspects of caring (humanitarian aspects, technical skills, knowledge, safety) as these aspects are expressed by nurses and expected by patients [40]. Thus, patient needs remain unmet, and this leads to patient dissatisfaction with the nursing care provided. However, these differences are to some extent expected. People in society come from various socio-economic classes, with different experiences and their own philosophy. Instead, nurses have received training and, in addition, think and act based on their experiences in the clinical setting and the environment in which they provide care. These differences are far from contradicting each other. Rather, they are complementary [41].

The major strength of this study is that it is the first study conducted in Greece using the 16-item version of the scale. Moreover, the sample comes from six large general hospitals in Greece (which are located in the country’s capital, the co-capital, and other large cities) spread over a fairly large area of mainland Greece. A limitation is that the questionnaires were distributed during the period of the pandemic COVID-19 and given the situation prevailing in the hospitals, the responses of the participants may be affected. In addition, the 11-year difference between the two groups could impact the perceptions between the groups.

## 5. Conclusions

The most important finding of the study is that there is a lack of agreement between patients’ and nurses’ perceptions of caring. It seems that nurses consider most important those caring behaviors related to the respect, privacy, and dignity of the patient. Instead, patients are more focused on behaviors, such as professional knowledge and skills to perform nursing activities and safety. Patients’ needs and perceptions should be considered in every strategic decision regarding nurse staffing. Understanding the differences will allow the gap that may exist to be bridged, so that the nursing care provided ensures patient satisfaction. For this reason, it is necessary to identify innovative approaches to resolve this lack of homogeneity and provide truly person-centered care.

## Figures and Tables

**Table 1:** Patients’ demographic and clinical characteristics (N = 310).

	Frequency	Percentage (%)
Gender		
Female	153	46.5
Male	157	47.7
Educational status		
Primary School	98	29.8
Secondary/High School	142	43.2
University	55	16.7
MSc/P.h. D	13	4
Marital status		
Married	221	67.2
Unmarried	42	12.8
Divorced	20	6.1
Widowed	27	8.2
Working Status		
Unemployed	48	14.6
Private employee	76	23.1
State employee	45	13.7
household	20	6.1
Retired	115	35
Student	6	1.8
Sector of Hospital		
Internal Medicine	232	70.5
Surgical Sector	76	23.1
Reason for admission		
Chronic disease	174	52.9
Acute disease	133	40.4
	Mean (SD)	Min–Max
Age (years)	60.8 (±16.8)	18–82
Duration of hospitalization (days)	11.62 (9.8)	3–60

SD: Standard Deviation.

**Table 2:** Nurses’ sociodemographic characteristics (Ν = 329).

Demographic Data	Frequency (%)	Percentage (%)
Gender		
Female	251	21.6
Male	71	76.3
Educational status		
High School	71	21.6
University	201	61.1
MSc/P.h.D	53	16.1
Marital status		
Married	190	57.8
Unmarried	92	28
Divorced	29	8.8
Widowed	9	2.7
Working department		
Internal medicine	130	39.5
Surgical department	113	34.3
Intensive Care Unit	22	6.7
Emergency Department	26	7.9
Operating theater	26	7.9
Other	7	2.1
Working position		
Nurse assistant	72	21.9
Nurse	229	69.6
Head nurse	20	6.1
Section Manager	3	0.9
Night shifts per month		
≤3	86	26.1
4–6	119	36.2
7–9	40	12.2
≥	7	2.1
	Mean (SD)	Min–Max
Age (years)	49 (±8.10)	23–60
Total experience as clinical nurse	17.6 (±8.5)	1–36

SD: Standard Deviation.

**Table 3:** Mean scores and ranking of CBI-16 items between the patients’ and nurses’ groups.

CBI-16 Items	Patients Mean ± SD	Ranking	Nurses Mean ± SD	Ranking
Attentively listening to me/the patient	5.09 ± 1.17	6	5.25 ± 0.82	4
Giving instructions or teaching me/the patient	5.00 ± 1.11	7	4.97 ± 0.94	12
Treating me/the patient as an individual	4.55 ± 1.46	15	5.32 ± 0.88	2
Spending time with me/the patient	4.69 ± 1.22	11	4.74 ± 0.94	15
Supporting me/the patient	4.79 ± 1.33	10	5.05 ± 3.07	10
Being empathetic or identifying with me/the patient	4.64 ± 1.49	12	5.17 ± 2.90	5
Being confident with me/the patient	5.17 ± 2.03	4	5.08 ± 2.45	8
Demonstrating professional knowledge and skill	5.45 ± 3.62	1	5.10 ± 0.94	7
Including me/the patient in planning care	4.36 ± 1.56	16	5.04 ± 5.38	11
Treating my/patient’s information confidentially	5.34 ± 1.06	2	5.43 ± 0.94	1
Returning to me/the patient voluntarily	4.59 ± 1.36	14	4.57 ± 3.68	16
Talking with me/the patient	4.62 ± 1.31	13	4.77 ± 1.09	14
Meeting my/patient’s stated and unstated needs	4.96 ± 3.71	8	4.86 ± 0.83	13
Responding quickly when I/the patient call	4.91 ± 1.12	9	5.07 ± 0.86	9
Giving my/patient’s treatments and medications on time	5.32 ± 0.99	3	5.26 ± 0.88	3
Relieving my/patient’s symptoms	5.12 ± 1.10	5	5.11 ± 0.93	6

SD: Standard Deviation.

**Table 4:** Differences in the mean values of patients and nurses.

	CBI-16 Items	Patients Mean ± SD	Nurses Mean ± SD	Mean Difference	95% CI		*t*-Test	df	*p*
					Lower	Upper			
Pair 1	Attentively listening to me/the patient	5.09 ± 1.17	5.25 ± 0.82	−0.16	−0.00	−0.00	−2.05	308	0.04
Pair 2	Giving instructions or teaching me/the patient	5.00 ± 1.11	4.97 ± 0.94	0.02	−0.13	0.19	0.34	306	0.72
Pair 3	Treating me/the patient as an individual	4.55 ± 1.46	5.32 ± 0.88	−0.76	−0.96	−0.57	−7.82	298	**0.00**
Pair 4	Spending time with me/the patient	4.69 ± 1.22	4.74 ± 0.94	−0.04	−0.22	0.12	−0.55	306	0.58
Pair 5	Supporting me/the patient	4.79 ± 1.33	5.05 ± 3.07	−0.25	−0.64	0.12	−1.31	295	0.18
Pair 6	Being empathetic or identifying with me/the patient	4.64 ± 1.49	5.17 ± 2.90	−0.52	−0.89	−0.15	−2.80	301	**0.00**
Pair 7	Being confident with me/the patient	5.17 ± 2.03	5.08 ± 2.45	0.09	−0.26	0.44	0.50	303	0.61
Pair 8	Demonstrating professional knowledge and skill	5.45 ± 3.62	5.10 ± 0.94	0.34	−0.07	0.77	1.62	302	0.10
Pair 9	Including me/the patient in planning care	4.36 ± 1.56	5.04 ± 5.38	−0.68	−1.33	−0.04	−2.10	304	0.36
Pair 10	Treating my/patient’s information confidentially	5.34 ± 1.06	5.43 ± 0.94	−0.09	−0.24	0.06	−1.16	304	0.24
Pair 11	Returning to me/the patient voluntarily	4.59 ± 1.36	4.57 ± 3.68	0.02	−0.41	0.45	0.10	305	0.91
Pair 12	Talking with me/the patient	4.62 ± 1.31	4.77 ± 1.09	−0.14	−0.34	0.44	−1.51	308	0.13
Pair 13	Meeting my/patient’s stated and unstated needs	4.96 ± 3.71	4.86 ± 0.83	0.09	−0.34	0.53	0.44	304	0.66
Pair 14	Responding quickly when I/the patient call	4.91 ± 1.12	5.07 ± 0.86	−0.15	−0.31	−0.00	−2.01	306	**0.04**
Pair 15	Giving my/patient’s treatments and medications on time	5.32 ± 0.99	5.26 ± 0.88	0.06	−0.09	0.21	0.79	307	0.42
Pair 16	Relieving my/patient’s symptoms	5.12 ± 1.10	5.11 ± 0.93	0.01	−0.15	0.18	0.19	306	0.84

CI: Confidence Interval, SD: Standard Deviation. The bold *p*-values are the statistically significant.

## References

[1] Bates R., Memel J.G. (2021). Florence Nightingale and responsibility for healthcare in the home. Eur. J. Hist. Med. Health.

[2] International Council of Nurses (2021). The ICN Code of Ethics for Nurses.

[3] Arthur D., Randle J. (2007). The professional self-concept of nurses: A review of the literature. Austrian J. Adv. Nurs..

[4] Ekpenyong S.M., Nyashanu M., Ossey-Nweze C., Serrant L. (2021). Exploring the perceptions of dignity among patients and nurses in hospital and community settings: An integrative review. J. Res. Nurs..

[5] Kruijver I.P.M., Kerkstra A., Bensing J.M., Van de Miel B.M.H. (2000). Nurse-patient communication in cancer care—A review of the literature. Cancer Nurs..

[6] O’Reilly L., Cara C. (2010). “Etre avec” la personne soignée en réadaptation: Une rencontre humaine profonde, thérapeutique et transformatrice [“Being with” the person cared for in a rehabilitation context: A profound, therapeutic and transformative human relationship]. Rech. Soins Infirm..

[7] Kelly M.A., Nixon L., McClurg C., Scherpbier A., King N., Dornan T. (2018). Experience of Touch in Health Care: A Meta-Ethnography Across the Health Care Professions. Qual. Health Res..

[8] Weger H., Bell G.C., Minei E.M. (2014). The Relative Effectiveness of Active Listening in Initial Interactions. Int. J. Listening.

[9] Doherty M., Thompson H. (2014). Enhancing person-centred care through the development of a therapeutic relationship. Br. J. Community Nurs..

[10] Chan E.A., Tsang P.L., Ching S.S.Y., Wong F.Y., Lam W. (2019). Nurses’ perspectives on their communication with patients in busy oncology wards: A qualitative study. PLoS ONE.

[11] Gunawan J., Aungsuroch Y., Watson J., Marzilli C. (2022). Nursing administration: Watson’s theory of human caring. Nurs. Sci. Q..

[12] Watson J. (2008). Nursing: The Philosophy and Science of Caring, revised ed..

[13] Jasemi M., Valizadeh L., Zamanzadeh V., Keogh B. (2017). A concept analysis of holistic care by hybrid model. Indian J. Palliat. Care.

[14] Baldursdottir G., Jonsdottir H. (2002). The importance of nurse caring behaviors as perceived by patients receiving care at an emergency department. Heart Lung.

[15] Vujanić J., Mikšić Š., Barać I., Včev A., Lovrić R. (2022). Patients’ and nurses’ perceptions of importance of caring nurse-patient interactions: Do they differ?. Healthcare.

[16] Cheruiyot J.C., Brysiewicz P. (2019). Nurses’ perceptions of caring and uncaring nursing encounters in inpatient rehabilitation settings in South Africa: A qualitative descriptive study. Int. J. Afr. Nurs. Sci..

[17] Rchaidia L., Dierckx de Casterlé B., De Blaeser L., Gastmans C. (2009). Cancer patients’ perceptions of the good nurse: A literature review. Nurs. Ethics.

[18] Karlou C., Papathanassoglou E., Patiraki E. (2015). Caring behaviours in cancer care in Greece. Comparison of patients’, their caregivers’ and nurses’ perceptions. Eur. J. Oncol. Nurs..

[19] Alikari V., Fradelos E.C., Papastavrou E., Alikakou S., Zyga S. (2021). Psychometric properties of the Greek version of the Caring Behaviors Inventory-16. Cureus.

[20] Wolf Z.R., Dillon P.M. (2017). Caring Behaviors Inventory-24 Revised: CBI-16 Validation and psychometric properties. Int. J. Hum. Caring.

[21] Wolf Z.R., Giardino E.R., Osborne P.A., Ambrose M.S. (1994). Dimensions of nurse caring. Image J. Nurs. Sch..

[22] Alquwez N., Cruz J.P., Al Thobaity A., Almazan J., Alabdulaziz H., Alshammari F., Albloushi M., Tumala R., Albougami A. (2021). Self-compassion influences the caring behaviour and compassion competence among Saudi nursing students: A multi-university study. Nurs. Open.

[23] Ferede A.J., Erlandsson K., Gezie L.D., Geda B., Wettergren L. (2022). Psychometric properties of the Caring Behaviors Inventory-16 in Ethiopia. Nurs. Rep..

[24] Ghafouri R., Nasiri M., Atashzadeh-Shoorideh F., Tayyar-Iravanlou F., Rahmaty Z. (2021). Translation and validation of Caring Behaviors Inventory among nurses in Iran. PLoS ONE.

[25] Papastavrou E., Karlou C., Tsangari H., Efstathiou G., Sousa V.D., Merkouris A., Patiraki E. (2011). Cross-cultural validation and psychometric properties of the Greek version of the Caring Behaviors Inventory: A methodological study. J. Eval. Clin. Pract..

[26] Poreddi V., Narayanan A., Thankachan A., Joy B., Awungshi C., Reddy S.S. (2021). Professional and ethical values in nursing practice: An Indian perspective. Investig. Educ. Enferm..

[27] Poorchangizi B., Borhani F., Abbaszadeh A., Mirzaee M., Farokhzadian J. (2019). The importance of professional values from nursing students’ perspective. BMC Nurs..

[28] Babaii A., Mohammadi E., Sadooghiasl A. (2021). The meaning of the empathetic nurse–patient communication: A qualitative study. J. Patient Exp..

[29] Pratt H., Moroney T., Middleton R. (2021). The influence of engaging authentically on nurse-patient relationships: A scoping review. Nurs. Inq..

[30] Wune G., Ayalew Y., Hailu A., Gebretensaye T. (2020). Nurses to patients communication and barriers perceived by nurses at Tikur Anbessa Specilized Hospital, Addis Ababa, Ethiopia 2018. Int. J. Afr. Nurs. Sci..

[31] Mizuno M., Ozawa M., Evans D., Okada A., Takeo K. (2005). Caring behaviours perceived by nurses in a Japanese hospital. J. Nurs. Stud..

[32] Alikari V., Tsironi M., Matziou V., Tzavella F., Stathoulis J., Babatsikou F., Fradelos E., Zyga S. (2019). The impact of education on knowledge, adherence and quality of life among patients on haemodialysis. Qual. Life Res..

[33] Zamanzadeh V., Azimzadeh R., Rahmani A., Valizadeh L. (2010). Oncology patients’ and professional nurses’ perceptions of important nurse caring behaviors. BMC Nurs..

[34] Poirier P., Sossong A. (2010). Oncology patients’ and nurses’ perceptions of caring. Can. Oncol. Nurs. J..

[35] Papastavrou E., Efstathiou G., Tsangari H., Suhonen R., Leino-Kilpi H., Patiraki E., Karlou C., Balogh Z., Palese A., Tomietto M. (2012). A cross-cultural study of the concept of caring through behaviours: Patients’ and nurses’ perspectives in six different EU countries. J. Adv. Nurs..

[36] Tucket A., Hughes K., Schluter P., Turner C. (2009). Validation of CARE-Q in residential aged-care: Rating of importance of caring behaviors from an e-cohort sun-study. J. Clin. Nurs..

[37] Sarabia-Cobo C., Sarriá E. (2021). Satisfaction with caregiving among informal caregivers of elderly people with dementia based on the salutogenic model of health. Appl. Nurs. Res..

[38] Gürcan M., Atay Turan S. (2021). Examining the expectations of healing care environment of hospitalized children with cancer based on Watson’s theory of human caring. J. Adv. Nurs..

[39] Thomas D., Newcomb P., Fusco P. (2019). Perception of caring among patients and nurses. J. Patient Exp..

[40] Oluma A., Abadiga M. (2020). Caring behavior and associated factors among nurses working in Jimma University specialized hospi-286 tal, Oromia, Southwest Ethiopia. BMC Nurs..

[41] Kieft R.A., de Brouwer B.B., Francke A.L., Delnoij D.M. (2014). How nurses and their work environment affect patient experiences of the quality of care: A qualitative study. BMC Health Serv. Res..

